# Epidermal Pulley Sutures and Double Burow’s Full-Thickness Skin Grafts for Large, High-Tension Closures

**DOI:** 10.7759/cureus.29122

**Published:** 2022-09-13

**Authors:** Brett C Neill, Jonny Hatch, Stanislav N Tolkachjov

**Affiliations:** 1 Dermatology, Oregon Health & Science University, Portland, USA; 2 Texas College of Osteopathic Medicine, University of North Texas Health Science Center, Fort Worth, USA; 3 Mohs & Complex Facial Reconstructive Surgery, Epiphany Dermatology, Dallas, USA

**Keywords:** extremity, skin cancer, skin surgery, pulley sutures, wound tension, reconstruction, mohs micrographic surgery, burow's graft

## Abstract

Large defects in high-tension areas can be difficult to close with primary closures alone. Skin grafts are often used. We describe a technique using epidermal pulley sutures and a Burow’s full-thickness skin graft (FTSG) that allows reconstruction of large defects while optimizing wound coverage and healing.

## Introduction

Partial closure followed by healing by secondary intention is often used for large defects that are subjected to high tension. When primary closures or flaps cannot completely close a defect, grafts can be used to cover the remaining exposed tissue. A Burow’s full-thickness skin graft (FTSG) is preferable due to adjacent tissue matching and avoidance of donor site morbidity [1]. Some Burow’s FTSGs do not fully cover the exposed tissue in large and/or high-tension areas, thus preventing appropriate sewing of the edges and require areas of granulation.

In addition, grafts must be in good contact with the wound bed to ensure graft survival. Without proper wound bed preparation and contact, the wound is at an increased risk of infection or graft failure [2]. Large grafts are more difficult to maintain proper graft and wound bed contact because the sutures on the periphery of the graft do not properly secure the center of the graft to the wound bed, and FTSG on the extremities may have lower rates of survival when compared to the head and neck. We describe a method for closing large, high-tension defects utilizing two Burow’s FTSGs and pulley sutures.

## Technical report

**Figure 1:**
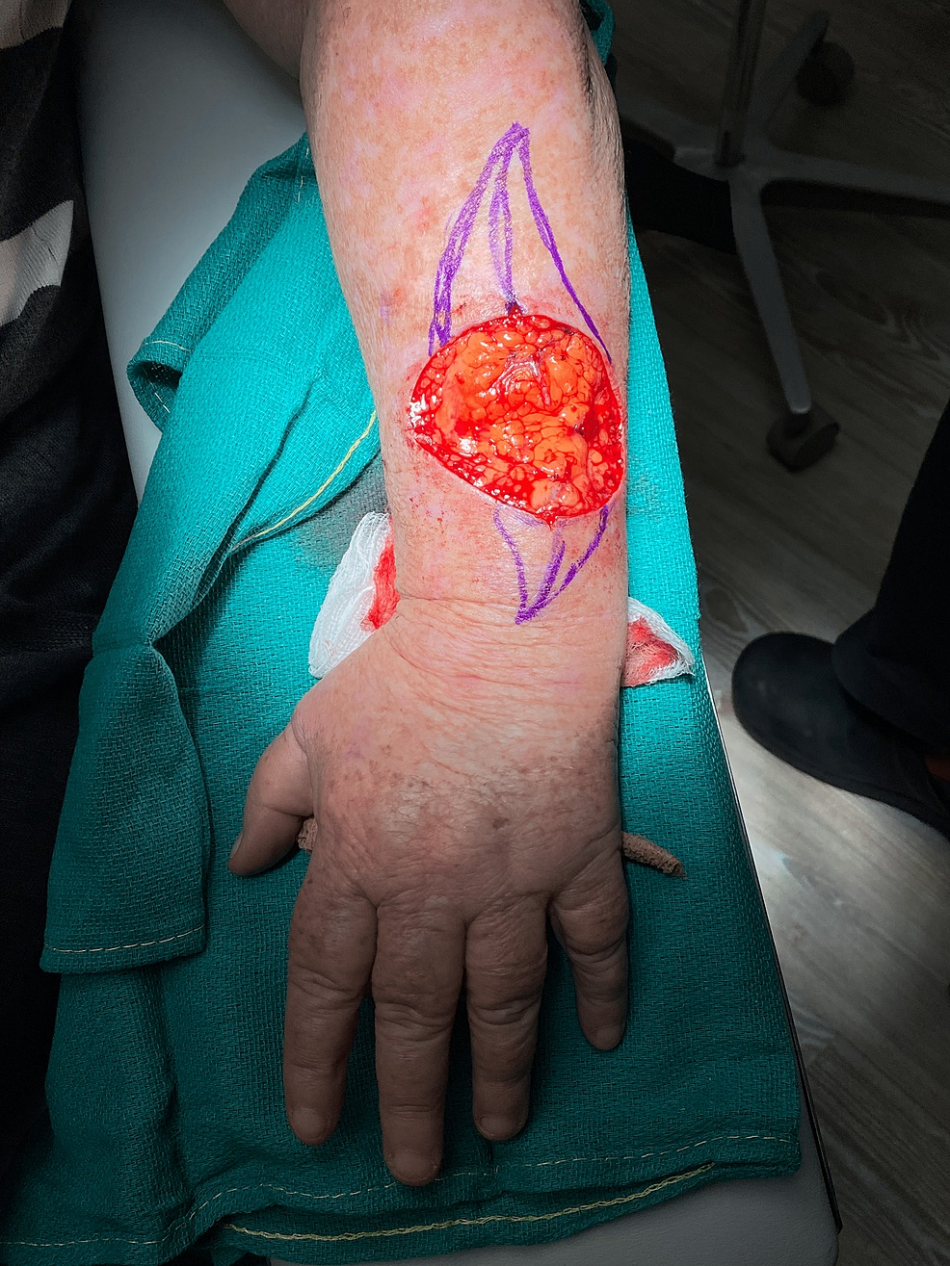
Large defect of the left forearm with Burow’s triangles outlined.

First, the Burow’s triangles are removed to facilitate a partial primary closure (Figure 1). Next, epidermal pulley sutures are placed about 0.5-1 cm apart and used to bring wound edges closer together, reducing the size of the defect. This is done by placing two adjacent epidermal stitches, without locking, and allowing the sliding of the suture knot after the second throw to act like a pulley system prior to locking.

**Figure 2:**
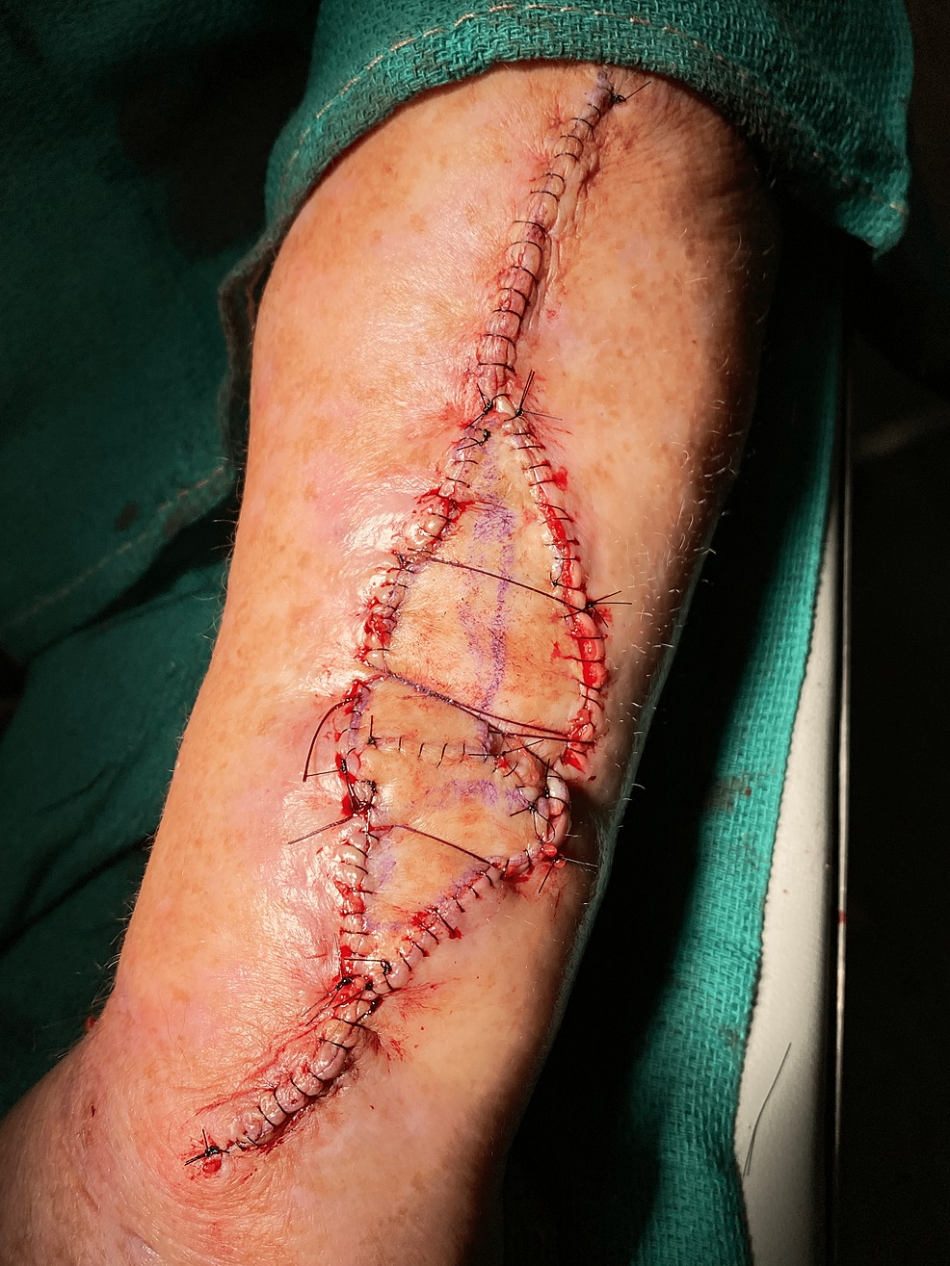
Large defect with two Burow’s FTSGs placed underneath the pulley sutures. Partial suturing of the FTSG may be employed as needed. Of note, only the central stitch had a pulley function in this case. FTSG, full-thickness skin graft

After proper graft and wound bed preparation, the two Burow’s FTSG are placed underneath the pulley sutures with the bases of the triangles adjacent to each other. In addition to shrinking the defect size, the pulley sutures promote graft and wound bed contact (Figure 2) which is essential for graft survival. A compressive wound dressing with petrolatum gauze is firmly applied to avoid potential sliding that could displace the grafts.

**Figure 3:**
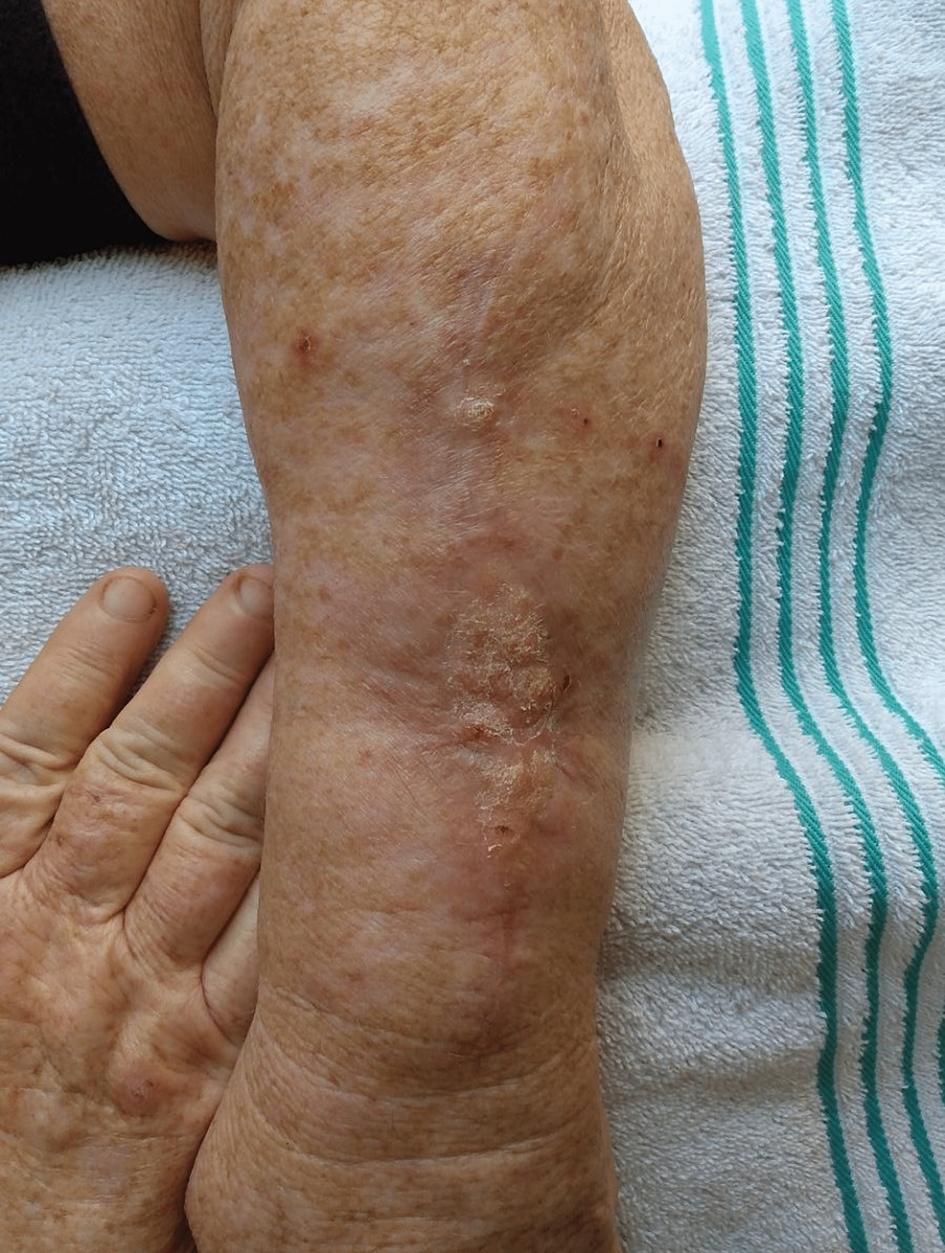
Excellent graft survival and wound healing five months after surgery. Of note, the patient was prone to rubbing the area, resulting in a lichen simplex chronicus appearance of the graft sites.

On follow up, the patient showed excellent graft survival and wound healing (Figure 3). The pulley sutures were successful in both approximating wound edges and helping with contact between the graft and the wound bed, avoiding the need for additional basting or tie-over sutures.

## Discussion

Large high-tension defects of the trunk and extremities can be difficult to close. The use of Burow’s triangles placed beneath pulley sutures simultaneously decreases the size of graft needed and encourages proper contact of the graft and the wound bed. A recent study conducted by Marsidi et al. attempted to determine the influence of different tie-overs and dressings on graft take for FTSGs [3]. The study suggests that regardless of technique used, graft survival rate is high. Various methods have been used to secure grafts to wound beds including tie-over bolsters [4], basting sutures [5], hydrocolloid foam [6], and surgical sponges [7]. Choice of graft fixation is determined by surgical location and surgeon preference. Using pulley sutures to decrease dead space between wound bed and graft is another option for surgeons to consider as they close large wounds and/or wounds in high tension areas.

## Conclusions

Decreasing dead space between graft and wound bed increases the chance of graft survival in FTSGs. Using pulley sutures to both decrease defect size and provide downward pressure on the skin graft is an excellent option to close defects. Use of this technique will assist dermatologists as they close large wounds, defects from tumors that need to be monitored, or those in high tension areas. 
